# A friend in knee: CCN3 may inhibit osteoarthritis progression

**DOI:** 10.1007/s12079-017-0446-y

**Published:** 2018-01-13

**Authors:** Alex Peidl

**Affiliations:** Department of Physiology and Pharmacology, University of Western Ontario, 1151 Richmond Street, London, Ontario, Canada

**Keywords:** CCN3, Extracellular matrix, Articular cartilage, Osteoarthritis, CCN proteins

## Abstract

Osteoarthritis (OA) is a major clinical problem among the ageing population, yet no disease-modifying treatments currently exist. This issue arises, in part, due to the complex processes occurring in the microenvironment of articular cartilage that lead to osteoarthritic changes. Gaining a better understanding of these processes is crucial in developing a viable therapy for OA. A recent report in Journal of Bone Mineral Metabolism by Janune et al. (J Bone Miner Metab 35:582–597, 2016) suggests a novel role for CCN3 in maintaining the differentiated phenotype of articular cartilage. This report suggests that CCN3, a member of the CCN family of matricellular proteins, is important for proteoglycan accumulation, as well as expression of type II collagen, tenascin C, and lubricin in vitro. Furthermore, exogenous CCN3 increased tidemark integrity and lubricin protein expression in a rat model of OA. These results implicate the regulation of CCN3 as a potential therapeutic target in patients with OA.

Early phases of osteoarthritis (OA) development often involve re-initiation of endochondral ossification, a process that occurs in embryos and children but ceases into adulthood, resulting in degradation of articular cartilage and osteophyte formation (Saito et al. 2010). Articular cartilage is thin hyaline cartilage that lines the joint surface of bones and plays a key role in maintaining a healthy joint. Currently, there is a lack of information on factors governing the regulatory processes that maintain the healthy articular cartilage phenotype, as well as factors that cause re-initiation of endochondral ossification in osteoarthritic cartilage. Investigating the microenvironment of articular cartilage may shed some light on these topics.

The CCN family of matricellular proteins (CCN1–6) play a crucial role in several biological processes including angiogenesis, cell proliferation, wound healing and tumorigenesis (Perbal 2013). Furthermore, these proteins are important for cartilage metabolism. CCN2, one of the most well-studied CCN proteins, has been shown to display an ability to regenerate articular cartilage (Nishida et al. 2007; Abd El Kader et al. 2014). CCN3, which has been shown to function reciprocally to CCN2 in a number of biological processes, activates the expression of genes related to articular chondrocyte differentiation, but represses proliferation of chondrocytes in costal growth plates (Janune et al. 2011). Furthermore, it has been shown that osteoarthritic changes occur in articular cartilage of mice lacking CCN3 protein, suggesting that CCN3 may play a role in the underlying mechanisms of OA pathogenesis (Roddy and Boulter 2015).

Given the importance of CCN3 in the microenvironment of articular cartilage, Janune et al. (2016) sought to uncover more about the potential role of CCN3 in the progression of OA. The authors aimed to investigate the reciprocal regulation of CCN3 during osteoarthritic changes in a monoiodoacetic acid (MIA)-induced model of OA, as well as evaluate the in vitro effects of CCN3 overexpression on articular chondrocytes. Furthermore, they wanted to investigate the in vivo effects of exogenous CCN3 on MIA-induced OA.

Results of the study showed that CCN3 is down-regulated in knee cartilage of rats with MIA-induced OA, compared to normal healthy rats, suggesting that CCN3 down-regulation is involved in the articular cartilage changes seen in this model of OA. The authors then used a Tet-on expression system to stably overexpress human CCN3 in articular chondrocytes in vitro. Overexpression of human CCN3 decreased chondrocyte proliferation, but also caused increased collagen type II (ECM component), lubricin (important for joint lubrication) and tenascin-C (associated with articular cartilage development) gene expression. Proteoglycan accumulation was also increased in CCN3-expressing chondrocytes.

Collectively, these results suggest that CCN3 may play an important role in maintaining the differentiated phenotype of articular chondrocytes in vitro. This action was then confirmed by the authors using in vivo studies. The authors used a gelatin hydrogel-mediated delivery of exogenous CCN3 into an MIA-induced rat model of OA and found that CCN3 treatment caused a slight decrease in the degeneration score of knee articular cartilage, although significance was not achieved. The CCN3-treated knees also appeared to have prominently broader tidemarks compared to the untreated groups, a positive outcome in OA progression. Finally, in CCN3-treated knees, lubricin appeared to be more abundant and localized to the surface of the articular cartilage, compared to the non-treated group. These results suggest that CCN3 treatment may protect against damage occurring to the articular cartilage surface in the early stages of OA.

The aforementioned study has revealed a great deal regarding the role of CCN3 in matrix metabolism of articular chondrocytes. Given the multi-modular structure of CCN proteins, future studies could be aimed at isolating the specific modules or peptide domains of CCN3 that may be important for these functions. Furthermore, assessing the role of CCN3 in other models of OA would provide additional evidence supporting the role of CCN3 in OA progression. Nonetheless, this study by Janune et al. (2016) is consistent with the notion that CCN3 or CCN3-derived peptides could be used in the future as a potential therapy for OA.
